# Genetic variants in *TMPRSS2* influence SARS-CoV-2 infection susceptibility within Mexican Mestizos

**DOI:** 10.3389/fgene.2025.1558189

**Published:** 2025-04-14

**Authors:** Rebeca I. Montero, Cinthia L. Dionicio, Gino Noris, Maricela Piña-Pozas, Carla Santana, Rocío Gómez

**Affiliations:** ^1^ Biología Molecular Diagnóstica, Querétaro, Qro, Mexico; ^2^ Secretaría de Salud, Iguala, Gro, Mexico; ^3^ Centro de Información para Decisiones en Salud Pública, Instituto Nacional de Salud Pública (INSP), Mexico City, Mexico; ^4^ Centro Cochrane Asociado al INSP, Mexico City, Mexico; ^5^ Departamento de Toxicología, Centro de Investigación y de Estudios Avanzados del Instituto Politécnico Nacional, Mexico City, Mexico

**Keywords:** TMPRSS2, gene polymorphism, rs4303795, rs8134378, rs75603675, SARS-CoV-2 infection, COVID-19

## Abstract

Since host genetics is one of the primary factors contributing to COVID-19 susceptibility and its clinical progression, several studies have focused on analysing the implications of genetic polymorphisms associated with COVID-19. These studies particularly emphasise on common variants in genes that are involved in the viral mechanism of host entry and in the host’s response to infection. In this study, we explored the participation of 24 single nucleotide polymorphisms located on the *ACE*, *ADAM17*, *FURIN*, *IFITM3*, *TMPRSS2* and *VDR* genes in SARS-CoV-2 infection susceptibility. Three of these SNPs in *TMPRSS2* (rs75603675, OR = 1.86, _95%_CI = 1.29–2.66, *p* ≤ 0.001; rs4303795, OR = 1.98, _95%_CI = 1.38–2.84, *p* ≤ 0.001 and rs8134378, OR = 2.59, _95%_CI = 1.28–5.21, *p* ≤ 0.01) had a significant association with an increased risk of infection. When comparing haplotype frequency distributions, the haplotypes CAG (OR = 7.34, _95%_CI = 5.51–9.77), AGA (OR = 2.46, _95%_CI = 1.12–5.44), and AGG (OR = 1.59, _95%_CI = 1.17–2.16) presented significant associations, suggesting that *TMPRSS2* influences SARS-CoV-2 infection susceptibility within Mexican Mestizos. These risk alleles and their haplotypes were found more frequently in the case group than in the reference group, contributing to at least a twofold increase in the risk of SARS-CoV-2 infection, a finding that was reinforced by meta-analyses.

## 1 Introduction

Severe acute respiratory syndrome coronavirus-2 (SARS-CoV-2) was responsible for thousands of deaths worldwide (Zhu et al., 2020; World Health Organisation, 2023). Although a wide range of clinical manifestations and severities (mild, severe and asymptomatic) have been reported, several factors have been associated with COVID-19 morbidity and mortality risk. Male sex and seniors (≥60 years old) have shown significant associations with morbidity and mortality (Hubacek, 2021; Shelton et al., 2021). Pre-existing medical comorbidities such as cardiometabolic disorders, kidney disease, malignancies, and neurodegenerative diseases have also been identified as critical factors influencing the severity of the symptoms and the likelihood of fatal outcomes (Ejaz et al., 2020; Huang et al., 2023).

The genetic background is a significant factor influencing susceptibility to COVID-19, its clinical course, and outcomes (Brest et al., 2020; Ishak et al., 2022; Ceylan, 2020). In this scenario, individuals of African ancestry have demonstrated the highest vulnerability to contracting COVID-19, whereas Asians and Europeans occupy second and third positions, respectively (Hubacek, 2021). Nonetheless, European populations such as Italians and Spanish people were severely affected, even more so than Asians (Ceylan, 2020; Gomez-Mesa et al., 2021; Farooqi et al., 2023). In Latin America, SARS-CoV-2 infection posed a significant public health challenge; this population with an admixed ancestry faced at least three viral infection waves (Gomez-Mesa et al., 2021; Santoro et al., 2022). According to data from March 2023, Mexico ranked 12th worldwide in confirmed COVID-19 cases, with over seven million reported cases (https://coronavirus.jhu.edu/map.html). Latinos, who exhibit notable susceptibility to the virus, serve as a genetic reservoir of these ancestral legacies (European, Asian, and African) (Sutton et al., 2022; Alvarez-Topete et al., 2024; Gomez et al., 2021; Santana et al., 2014). Latino populations are particularly young, with at most twenty generations. Consequently, their genetic architecture could present interethnic variations, creating an ideal context to explore the role of ethnic background in disease predisposition (Brest et al., 2020; Ishak et al., 2022).

Different polymorphic gene variants have been analysed concerning COVID-19 susceptibility, and several studies have focused on common variants associated with the risk of infection from other respiratory viruses (Cheng et al., 2015; David et al., 2022; Posadas-Sanchez et al., 2022). Additionally, other studies have highlighted the relationship between blood type and ancestry in the differential risk among populations (Shelton et al., 2021). In this context, the Genome Aggregation Database has reported that certain variants related to the severity of COVID-19 clinical manifestations exhibit high frequencies within European and African populations (Niemi et al., 2022). Despite the diverse genetic makeup of Latino populations, there is a limited number of studies addressing the influence of ethnic background and the distribution of the genetic variants in the function of SARS-CoV-2 infection susceptibility.

In the present study, we evaluated the association of 24 single nucleotide polymorphisms located in the *ACE2*, *ADAM17*, *FURIN*, *IFITM3*, *TMPRSS2*, and *VDR* genes with SARS-CoV-2 infection susceptibility in 489 Mexican Mestizos (case and reference groups) residing in Queretaro. Allele, genotype and haplotype analyses were conducted in our target population as in those populations that contributed to its genetic background; meta-analyses further supported our findings.

## 2 Materials and methods

### 2.1 Genes and single nucleotide polymorphisms selection

We selected 24 single nucleotide polymorphisms (SNPs) located in the *ACE2*, *ADAM17*, *FURIN*, *IFITM3*, *TMPRSS2*, and *VDR* genes (Supplementary Table S1). The selection of genes was based on previous reports regarding virus entry mechanisms, respiratory illnesses, and *in silico* experiments; all genetic variants exhibited frequencies ≥1% in the 1000 Genomes Project database (1KGP; https://www.internationalgenome.org/).

### 2.2 Studied groups characteristics

A convenience sampling cross-sectional study was conducted from August 2020 to July 2021 involving unrelated individuals (no parent, grandparent, sibling, uncle or cousin of the participants were involved) who visited the *Biología Molecular Diagnóstica* (BIMODI) Laboratory to undergo SARS-CoV-2 testing. BIMODI fullfilled all requirements to process SARS-CoV-2 samples as endorsed by the Institute of Diagnosis and Epidemiological Reference from Mexico (InDRE, acronym in Spanish). Inclusion criteria considered for both the case and reference groups were: males and females self-identified as Mexican Mestizos with four grandparents born in Mexico, who completed a questionnaire and signed an informed consent form. Pregnancy and breastfeeding were used as exclusion criteria because these physiological states alter the immune system, representing a possible change in susceptibility to infections. The cases were individuals who tested positive for SARS-CoV-2 identified through the analysis of nasopharyngeal swabs using real-time reverse transcription polymerase chain reaction (RT-PCR). A total of 241 adults were enrolled as cases, 107 men and 134 women; age mean and standard deviation: 40 ± 14 years (residing in Santiago de Querétaro, a Central Valley of Mexico state located 213 km northwest of Mexico City). In such individuals, the prevalence of COVID-19 comorbidities was assessed using a questionnaire. The population reference allele frequencies were determined from a DNA bank of individuals who visited the laboratory before the SARS-CoV-2 pandemic, reflecting the ethnic and socioeconomic characteristics of the population that BIMODI laboratory attends for routine clinical check-ups. This group was populational-based (124 men and 124 women) and consisted of unrelated individuals. Such particularities minimised potential bias related to increased resistance or susceptibility to SARS-CoV-2 infection, which could exist if never-infected individuals had been included as a control. Cases were paired by sex with the reference group.

An internal Ethics Committee composed of external researchers approved protocol BMD-DI-2020-001 and the sample collection. The present study was conducted in accordance with the principles established by the Declaration of Helsinki.

### 2.3 Sample collection and molecular analyses

Samples for SNP genotyping were obtained from peripheral blood leukocytes using the BD Vacutainer system (Becton Dickinson, Franklin Lakes, NJ, United States) using EDTA as anticoagulant and DNA was isolated from the buffy coat with QIAamp^®^ DNA Mini Kit (QIAGEN, Venlo, NLD) using the QIAcube automated sample preparation (QIAGEN, Venlo, NLD). In some cases, SNP genotyping was made from genomic material isolated from the nasopharyngeal swab taken for the SARS-CoV-2 test using Quick RNA Viral Kit (Zymo Research, Irvine, CA, United States).

Real-time Polymerase Chain Reaction (qPCR) was used to quantify the genetic material with a Quant Studio™ 7 Real-Time PCR System (Applied Biosystems, Waltham, MA, United States) using 348 plates following conditions recommended by the manufacturer. Allelic discrimination of SNPs was conducted by qPCR using Master kit GoTaq^®^ 1 Step RT-qPCR System (Promega, Madison, WI, United States), and TaqMan^®^ assays (Supplementary Table S1) (Applied Biosystems, Waltham, MA, United States) in the Quant Studio™ 7 Real-Time PCR System (Applied Biosystems, Waltham, MA, United States). The thermal cycling conditions for TaqMan^®^ assays were 95°C for 10 min, followed by 40 cycles of 95°C for 15 s, and 60°C for 1 min. The genotyping process was conducted in a double-blinded manner for the analyst.

### 2.4 Statistical analyses

#### 2.4.1 Genetic analyses

Allelic, genotypic, and haplotypic frequencies, as well as observed (Ho) and expected (He) heterozygosity, and the analysis of molecular variance (AMOVA) were estimated with Arlequin v3.5 (Excoffier and Lischer, 2010). Linkage disequilibrium (LD) likelihood estimations of the analysed SNPs was carried out using their genetic position and genotypes reported in the 1KGP database (https://www.internationalgenome.org/). The LD heat map was obtained using the online platform for data visualisation SRplot (Tang et al., 2023). Weir and Cockerham’s *F* statistics were used to determine the Hardy-Weinberg expectation (HWE) with Genètix v4.05.2 (Belkhir et al., 2004). Genetic distances (*F*_*ST*_) among the explored groups were also calculated with Arlequin v3.5 using 1,000 permutations and visualised with a multidimensional scaling (MDS) plot with the Statistical Package for Social Sciences (SPSS) v23 (IBM Corp., Armonk, NY, United States). All *p* values were adjusted for false discovery rates (FDR) (Benjamini and Hochberg, 1995). Haplotype networks were constructed using Median-Joining (MJ) with Populational Analysis with Reticulate Trees (PopART) (Leigh and Bryant, 2015).

#### 2.4.2 Comparison with other populations

The diversity patterns obtained were compared with those populations that historically contributed to the genetic background of the Mexican Mestizo. Data from African (AFR), East Asian (EAS), and European (EUR) populations, as well as Mexican Ancestry in Los Angeles, California (MXL) was obtained from the 1KGP database (https://www.internationalgenome.org/). Additionally, our data was also compared with other Latin American populations, including Colombians in Medellin (CLM), Puerto Ricans in Puerto Rico (PUR), and Peruvians in Lima (PEL).

#### 2.4.3 Associations with SARS-CoV-2 infection susceptibility

Case and reference groups were compared; depending on the variable type and distribution, Student’s t-test, χ^2^ test, or Fischer exact test were employed. The genetic association with SARS-CoV-2 infection susceptibility was estimated with an unconditional logistic regression model. The age at the time of the interview was included as a continuous variable. Measures of gene variant effects were determined through odds ratio (OR). Analyses were performed using statistical software STATA v14 (StataCorp LP, College Station, TX, United States). In those SNPs where associations with the SARS-CoV2 susceptibility were identified in allele and genotype comparisons, further analyses were conducted using dominant and recessive models.

#### 2.4.4 Genetic comparisons with previous reports: Meta-analyses of SARS-CoV-2 infection susceptibility

The effects of our findings were also combined with results obtained from other publications. Measures of gene variant effects were estimated by comparing frequency differences between cases and the reference group. Meta-analyses were performed with the web-based app MetaGenyo (Martorell-Marugan et al., 2017). Subgroup analyses were performed using the web-based app Meta-analysis made easy developed with Shiny using the R packages meta and metafor (Viechtbauer, 2010). Heterogeneity among the included studies and the data obtained from the present study was determined with the heterogeneity index (I^2^). Values greater than or equal to 25%, 50%, and 75% were classified as low, moderate and high heterogeneity, respectively. If heterogeneity was ≥25%, the random effects model was applied, and the DerSimonian and Laird method was used as the weight method; otherwise, the fixed effects model was used, and the inverse of variance was used as the weight method. All results were summarised with forest plots using the statistical Mantel-Haenszel method to analyse the effect size from the combined results of the various studies included.

## 3 Results

The case group exhibited the following prevalence of comorbidities relevant to COVID–19 development: diabetes (7%), hypertension (9%), cardiovascular diseases (2%), smoking (24%), obesity (24%), and asthma (2%). This population was distributed in the following age groups: 0–14 years (1%), 15–24 years (9%), 25–34 years (28%), 35–44 years (24%), 45–54 years (22%), >55 years (16%). The sex distribution was as follows: female (55%) and male (45%). The reference group was chosen to have the same sex distribution, but the distribution of its comorbidities is unknown.

The allele and genotype frequencies obtained from all analysed polymorphisms are shown in Table 1; Supplementary Table S2, S3. Individually and collectively, all SNPs exhibit Hardy-Weinberg equilibrium in cases (*F*_*IS*_ = −0.014, *p* = 0.826) and the reference group (*F*_*IS*_ = 0.044, *p* = 0.005) with a nuanced heterozygous and homozygous excess, respectively; the *p*-value applying Bonferroni’s correction was 0.003. AMOVA test exhibits an irrelevant variance (0.004) among populations (cases versus reference group), with the variation within populations as the main source of variance (0.996, *p* = 0.003).

**TABLE 1:** Allele, minimum allele, genotype frequencies and equilibrium regarding the Hardy-Weinberg equation in the seven *TMPRSS2* polymorphisms.

SNP	Case group (*n* = 241)	Reference group (*n* = 248)	MAF		
			MXL (*n* = 64)	EUR (*n* = 503)	EAS (*n* = 504)
rs456298 T>A g.41464824T>A					
T	190 (0.39)	201 (0.41)	0.625	0.831	0.364
A	292 (0.61)	295 (0.59)			
TT	39 (0.16)	46 (0.19)			
TA	112 (0.46)	109 (0.44)			
AA	90 (0.37)	93 (0.38)			
HWE *F*_*IS*_ (*p*)	0.029 (0.376)	0.090 (0.076)			
rs2070788 G>A g.41470061G>A					
G	240 (0.50)	264 (0.53)	0.531	0.536	0.644
A	242 (0.50)	232 (0.47)			
GG	59 (0.24)	75 (0.30)			
GA	112 (0.51)	114 (0.46)			
AA	60 (0.25)	59 (0.24)			
HWE *F*_*IS*_ (*p*)	−0.010 (0.643)	0.079 (0.154)			
rs61735794 C>T g.41470664C>T					
C	472 (0.98)	494 (0.99)	0.008	0.030	0
T	10 (0.02)	2 (0.00)			
CC	232 (0.96)	246 (0.99)			
CT	8 (0.03)	2 (0.00)			
TT	1 (0.004)	0 (0)			
HWE *F*_*IS*_ (*p*)	0.185 (0.104)	−0.002 (1)			
rs12329760 C>T g.41480570C>T					
C	408 (0.85)	425 (0.86)	0.180	0.236	0.362
T	74 (0.15)	71 (0.14)			
CC	173 (0.72)	184 (0.74)			
CT	62 (0.26)	57 (0.23)			
TT	6 (0.02)	7 (0.03)			
HWE *F*_*IS*_ (*p*)	0.012 (0.506)	0.065 (0.189)			
rs75603675 C>A g.41507982C>A					
C	337 (0.7)	399 (0.80)	0.281	0.405	0.017
A	145 (0.3)	97 (0.20)			
CC	114 (0.47)	155 (0.63)			
CA	109 (0.45)	89 (0.36)			
AA	18 (0.07)	4. (0.02)			
HWE *F*_*IS*_(*p*)	−0.077 (0.91)	−0.139 (0.997)			
rs4303795 A>G g.41508558A>G					
A	328 (0.68)	391 (0.78)	0.281	0.411	0.018
G	154 (0.32)	105 (0.21)			
AA	106 (0.44)	151 (0.61)			
AG	116 (0.48)	89 (0.36)			
GG	19 (0.08)	8 (0.03)			
HWE *F*_*IS*_(*p*)	−0.095 (0.952)	−0.073 (0.90)			
rs8134378 G>A g.41521831G>A					
G	454 (0.94)	484 (0.98)	0.023	0.115	0.001
A	28 (0.06)	12 (0.02)			
GG	213 (0.88)	236 (0.95)			
GA	28 (0.12)	12 (0.05)			
AA	0 (0)	0 (0)			
HWE *F*_*IS*_(*p*)	−0.059 (1)	−0.023 (1)			

EAS, East Asian populations; EUR, European populations; *F*_*IS*_, fixation index; MAF, minimum allele frequency; HWE, Hardy-Weinberg Equilibrium; MXL, Mexican ancestry in Los Angeles, California; *n*, number of samples; *p*, p-value.

### 3.1 *TMPRSS2* polymorphisms were associated with SARS-CoV-2 susceptibility

**FIGURE 1:**
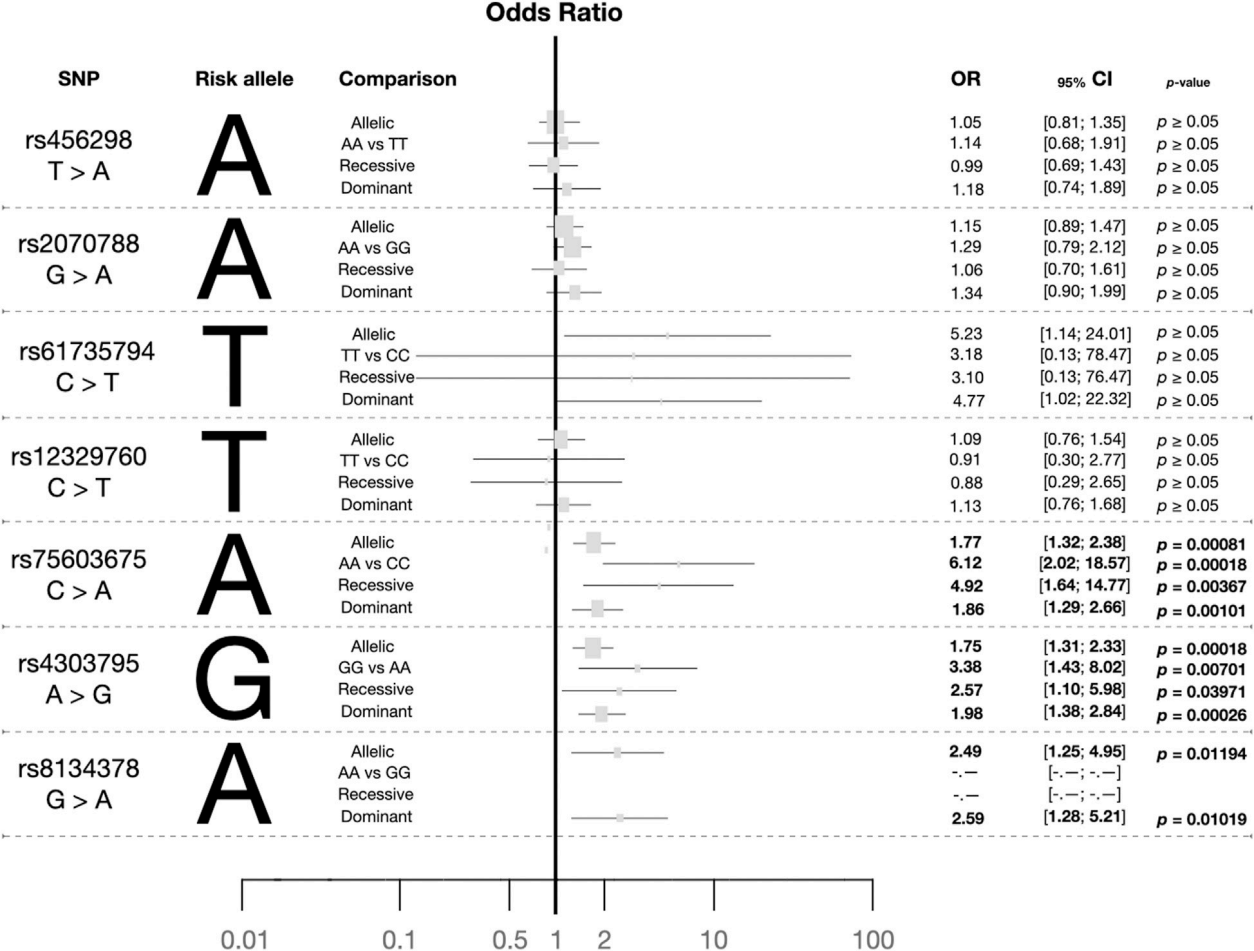
Plot summarising the risk alleles and the odds ratios obtained from allele and genotype comparisons between the case and reference groups for the seven *TMPRSS2* polymorphisms explored using several genotype models. Footnote: The dominant and recessive combinations conducted were: rs4562985_Dominant_ = AA + AT versus TT, rs4562985_Recessive_ = AA vs. AT + TT; rs20707885_Dominant_ = AA + AG vs. GG, rs20707885_Recessive_ = AA vs. AG + GG; rs61735794_Dominant_ = TT + TC vs. CC, rs61735794_Recessive_ = TT vs. TC + CC; rs12329760_Dominant_ = TT + TC vs. CC, rs12329760_Recessive_ = TT vs. TC + CC; rs75603675_Dominant_ = AA + AC vs. CC, rs75603675_Recessive_ = AA vs. AC + CC; rs4303795_Dominant_ = GG + GA vs. AA, rs4503795_Recessive_ = GG vs. GA + AA; rs8134378_Dominant_ = AA + AG vs. GG, rs8134378_Recessive_ = AA vs. AG + GG.

**FIGURE 2:**
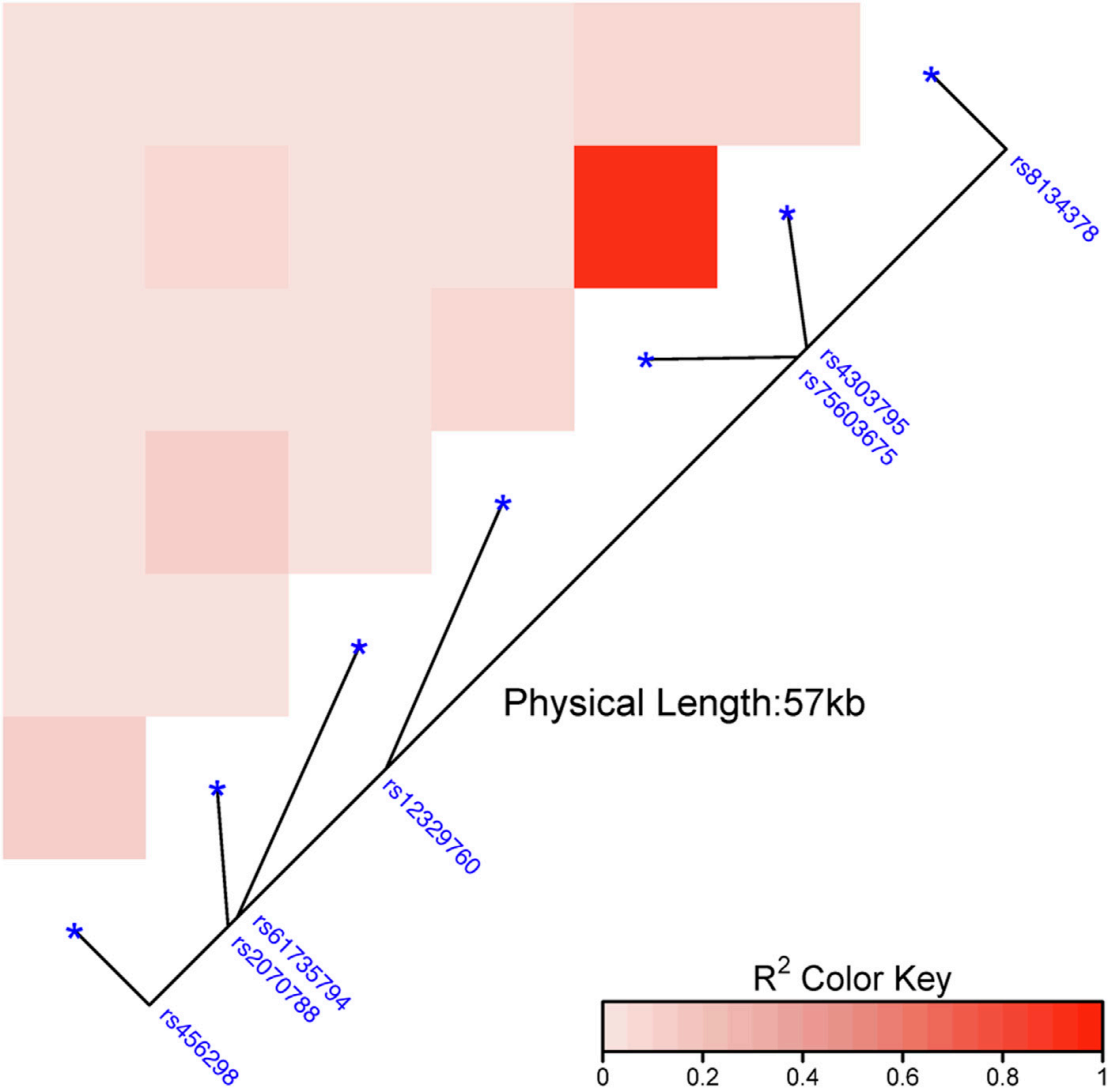
Linkage disequilibrium heatmap on the reference group based on the genetic distances among the several polymorphisms studied in *TMPRSS2* gene. **Note**: The intensity of the colours means the closeness between the SNPs and, in turn, their linkage disequilibrium.

Out of the 24 explored polymorphisms (Supplementary Table S1), only some of them in the *TMPRSS2* gene were associated with the risk of infection with SARS-CoV-2 (Figure 1; Supplementary Figure S1). Most of these SNPs were in LD (*p* ≤ 0.0001; Figure 2). Four of them (rs61735794, rs75603675, rs4303795, and rs8143478) were linked to an increased susceptibility to SARS-CoV-2 infection (Figure 1), exhibiting significant differences in allele and genotype frequencies between the case group and the reference one (Table 1). Nonetheless, the results for rs61735794 should be interpreted with caution due to the wide confidence interval and because the *p*-value was not significative (OR = 4.77; _95%_CI = 1.02–22.32, *p* ≥ 0.05); rs456298, rs2070788 and rs12329760 show marginal associations (Figure 1).

Thus, subsequent analyses focused on the three polymorphisms (rs75603675, rs4303795 and rs8134378) with a significant association; the alleles A, G, and A were identified as risk alleles, respectively. In the context of the genotypic state, specific distributions were observed (Table 1). The most common genotype of rs75603675 was CC in cases and the reference group. Regarding the frequency distribution of rs4303795, the heterozygous state (AG) was more prevalent in the case group (0.480) than in the reference group (0.360), where the AA genotype presented the main frequency (0.610). In cases and the reference group, the most common genotype for rs8134378 was GG. In terms of each variant´s infection risk using the random model (Figure 1), rs75603675 had the lowest association (OR = 1.86; _95%_CI = 1.29–2.66, *p* = 0.001), followed by rs4303795 (OR = 1.98; _95%_CI = 1.38–2.84, *p* ∼ 0.0003); and rs8134378 exhibited the highest association (OR = 2.59; _95%_CI = 1.28–5.21, *p* = 0.010).

### 3.2 Comparisons of *TMPRSS2* haplotypes between the selected populations

**FIGURE 3:**
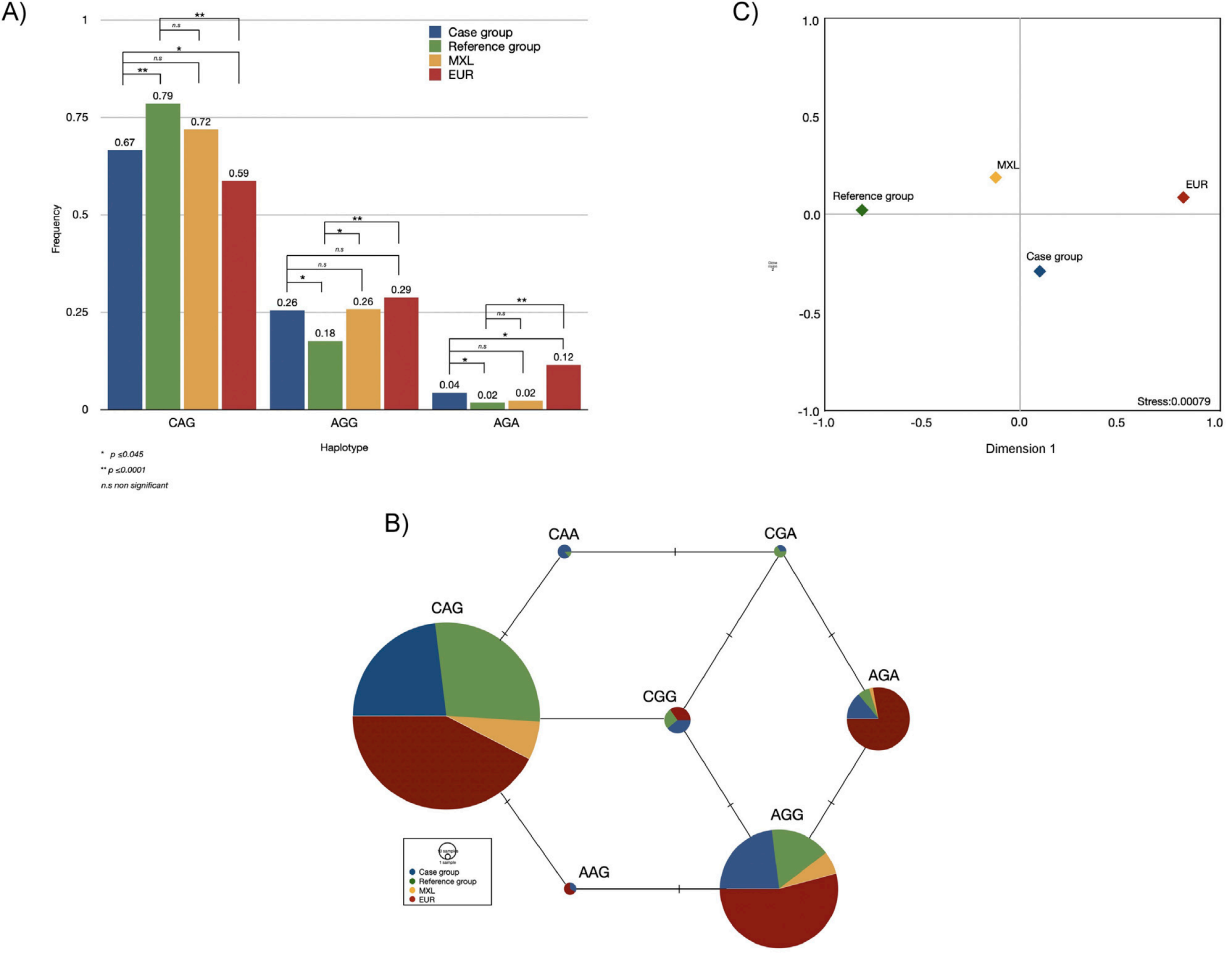
Bar plot with the statistical differences **(A)**, median-joining network **(B)**, and multidimensional scale plot of *F*_*ST*_ values **(C)**, of haplotypes found in the case and reference groups and their comparisons with two populations from the one thousand genome project. Note: MXL, Mexican in Los Angeles, California; EUR, European populations. The alleles of the SNPs conforming to the haplotype are shown in the following order: rs75603675, rs4303795, rs8134378.

Having demonstrated the contribution of these three SNPs (rs75603675, rs4303795, and rs8134378) and the LD among them, haplotypes were constructed. Such haplotypes are presented in the same SNPs’ order. Out of eight possible combinations, seven were identified within the analysed groups. Of these, the haplotype CAG (OR = 7.34; _95%_CI = 5.51–9.77, *p* < 0.0001) presented the strongest association, followed by AGA (OR = 2.46; _95%_CI = 1.12–5.44, *p* = 0.03), and AGG (OR = 1.59; _95%_CI = 1.17–2.16, *p* = 0.004). Such haplotypes were compared with those obtained from the 1KGP populations. Both cases and the reference group exhibited significant and no significant differences with EUR and MXL, respectively (Figure 3A). The MJ network show the remarkable prevalence of the CAG, AGG and AGA haplotypes across the compared populations (Figures 3A, B). It is important to highlight the remarkable prevalence of these haplotypes, as well as AAG, among Europeans, the high distribution of the CAA haplotype in the case group, and CGA in the studied groups. Comparisons with the remaining Latin American populations from 1KGP revealed a strong connection between MXL and PEL, although this data is not presented.

Although the case and reference groups exhibit a modest genetic distance to MXL (*F*_ST_ < 0.05), indicating that both groups belonged to the same population (*p* ≥ 0.05), significant differences were observed between cases and the reference group (*F*_ST_ = 0.2684, *p* ≤ 0.0001). Genetically, the case group was four times closer to EUR (*F*_ST_ = 0.01776) than the reference group (*F*_ST_ = 0.08148) and almost twice than MXL (*F*_ST_ = 0.03018). This finding was further supported by the MDS plot (stress value ∼0.0008), showing the genetic relationship between EUR and the case group, distinguishing them from the MXL and the reference group (Figure 3C). Nonetheless, the genetic distances between the studied groups and EUR were statistically significant (Supplementary Table S4).

### 3.3 Meta-analyses support *TMPRSS2* association with SARS-CoV-2 susceptibility

**FIGURE 4:**
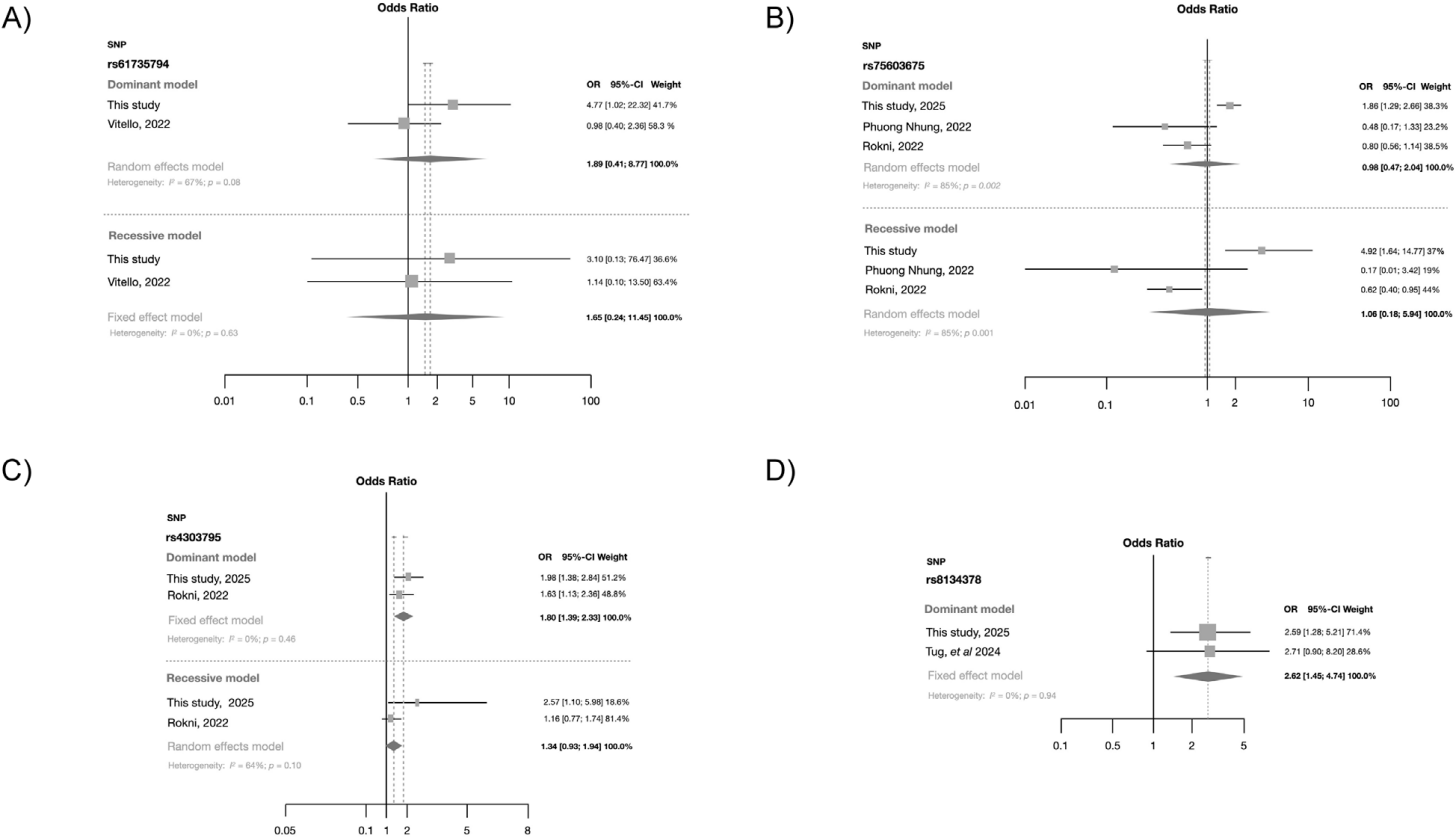
Forest plot obtained from the fixed effect model showing the risk contribution under the dominant and recessive models (when possible) in the four *TMPRSS2* polymorphisms with risk association: **(A)** rs61735794, **(B)** rs75603675, **(C)** rs4303795 and **(D)** rs8134378.

Our findings were compared with those reported from prior studies through forest plots using fixed and random effect models (Figure 4). The effects observed for rs61735794 and rs75603675 differ from those reported in earlier studies (Figures 4A, B). In summary, the forest plots indicate that these two polymorphisms had no significant association with SARS-CoV-2 infection susceptibility. In contrast, polymorphism rs4303795 (Figure 4C) exhibited a significant association with SARS-CoV-2 infection under both dominant (OR = 1.80; _95%_CI = 1.39–2.33) and recessive models, although the latter was marginal and exhibited high heterogeneity (*I* ^2^ = 64%). SNP rs8134378 also showed a significant association (OR = 2.62; _95%_CI = 1.45–4.74) under the dominant model (Figure 4D). Additional meta-analyses were conducted using the other *TMPRSS2* genetic variants; however, no associations were found (Supplementary Figures S2-5).

### 3.4 Other polymorphisms

Although previous studies have identified certain polymorphisms in the *ACE2*, *ADAM17*, *FURIN*, *IFITM3*, and *VDR* genes associated with infection susceptibility to SARS-CoV-2, our findings did not reveal any such associations (data not shown).

## 4 Discussion

COVID-19 results from the interplay of numerous genetic variants together with pre-existing medical comorbidities, individual precautions, hygiene practices, and even government-imposed restrictions (Hubacek, 2021; Sutton et al., 20 22). In particular, the risk of COVID-19 is increased for individuals living with comorbidities such as cardiometabolic and lung and kidney diseases (Camelo et al., 2024). Obesity and diabetes are significant risk factors that are prevalent within the Mexican population (Li et al., 2023). COVID-19 complications could increase in persons with cancer, immunosuppression, malnutrition and its consequences (Camelo et al., 2024; Villapalos-García et al., 2022). Further, male sex and advanced age (>65 years) are considered risk factors for a severe course (Lott et al., 2022). Lifestyle factors also contribute to susceptibility; cigarette smoking and avoiding hand-washing have been considered SARS-CoV-2 infection risk factors (Liu et al., 2023; Lott et al., 2022). In addition to the previous factors, the genetic ones are also relevant; individual genetic variability and gene interactions play a critical role in the differences observed in susceptibility to this and other infectious diseases. Host genetics has been one of the most accredited factors contributing to COVID-19 due to its association with susceptibility and clinical course (Brest et al., 2020). Several genes were studied during the pandemic for their association with COVID-19 clinical manifestations and outcomes, particularly those related to viral entry mechanisms (Yamamoto et al., 2021). Among these, *TMPRSS2* have been widely studied due to its role in SARS-CoV-2 membrane fusion to the host cell. Some gene polymorphisms seem to contribute to susceptibility and severity in some populations but not others, demonstrating that genetic background may play a role in COVID-19 development (Gupta and Misra, 2020). Latin American populations have experienced various demographic events over the past 500 years, providing an ideal context to investigate whether the characterisation of previously described gene variants is related to the susceptibility of infection and clinical manifestations and if this could be due to the ancestral background (Gomez et al., 2021). In the present study, 24 single nucleotide polymorphisms located on *ACE2*, *ADAM17*, *FURIN*, *IFITM3*, *TMPRSS2*, and *VDR* genes were characterised in 241 adults infected with SARS-CoV-2 (cases), along with their respective reference population paired by sex. However, because our cases and reference population were sampled at different times and came to BIMODI for different reasons, genetic background and environmental exposures could not be identical for both groups. The results were compared with findings from other studies using meta-analyses and multidimensional scale plots.

### 4.1 *TMPRSS2* polymorphisms association with SARS-CoV-2 infection susceptibility

Despite numerous studies demonstrating the role of *TMPRSS2* in the development of SARS-CoV-2, the current findings enhance the understanding of how these gene polymorphisms influence susceptibility to infection. Out of the seven analysed polymorphisms, three (rs75603675, rs4303795, and rs8134378) presented a significant association. Similar results have been reported for rs75603675 in another Mexican sample, as well as in Italian, Russian, and Spanish populations, thereby reinforcing our findings (Martínez-Gomez et al., 2024; Calcagnile et al., 2024; Minashkin et al., 2022; Torres-Fuentes et al., 2021; Villapalos-García et al., 2022). Besides its role in infection susceptibility, rs75603675 has been involved in symptomatology, clinical manifestations, severity, and mortality (Martínez-Gomez et al., 2024; Minashkin et al., 2022; Torres-Fuentes et al., 2021; Villapalos-García et al., 2022). TMPRSS2 is primarily expressed in the lungs, thyroid, and small intestinal epithelia, among other tissues. Different expression patterns of TMPRSS2 have been reported, mainly in lung tissue, the primary site of infection (Irham et al., 2020). Thyroid hormone levels play a role in coagulation, which is one of the leading causes of death associated with COVID-19; antithrombin, a coagulation regulator, inhibits TMPRSS2, thereby reducing the likelihood of coronavirus infection (Wettstein et al., 2023). Furthermore, TMPRSS2 has shown significantly higher expression in duodenum tissue from SARS-CoV-2 infected patients than uninfected individuals, which has been associated with gastrointestinal symptoms (Lin et al., 2023).

TMPRSS2 structure could be modified by missense variants such as rs75603675, whose allelic substitution (*c.*23C > A) provokes an amino acid change (*p*.Gly8Val). Even though both amino acids are nonpolar, there are significant differences between them. Glycine is the smallest amino acid and the only one with an achiral carbon which could provoke structural changes. *In-silico* studies have revealed that the substitution of Gly by Val, along with the modifications in other SNPs, might affect the N-terminal ends and, in turn, TMPRSS2 folding (Calcagnile et al., 2024; Paniri et al., 2021; Saih et al., 2021). The Human Splicing Finder (HSF), an *in silico* splice sites tool, predicted a broken donor splice site caused by substituting one allele with another in rs75603675 (Paniri et al., 2021). Furthermore, the number of hydrogen bonds is also different: ≈35 in TMPRSS2-Gly8 and more than 40 in TMPRSS2-Val8, conferring greater stability. In its ancestral form (rs75603675-C-allele-*p.*Gly), the endocytic signal is eclipsed by tyrosine at position 26, which partially covers it, causing the endocytic signal to fall in a flexible region (Minashkin et al., 2022). In contrast, when the derivate allele (rs75603675-A-allele-*p*.Val) is present, the endocytosis signal is more accessible, which translates to a higher susceptibility of the SARS-CoV-2 infection, supporting our findings as well as those of other research groups (Calcagnile et al., 2024). Similar findings have been reported for the intron variant rs8134378, which is in linkage disequilibrium with rs75603675 (Tug et al., 2024).

Polymorphism rs4303795, located in the promoter region, could modify TMPRSS2 expression. A previous report suggested that the derivative allele (rs4303795-G) could create binding sites for the Hepatocyte Nuclear Factor 4 (HNF4) and the Kidney, Ischaemia, and Developmentally-Regulated Protein 3 (KID3) (Vargas-Alarcon et al., 2020). Both HNF4 and KID3 orchestrate various transcriptional programs in different organs, and their dysregulation is associated with chronic inflammatory states, which are central to COVID-19 symptomatology (Vemuri et al., 2023; Jung et al., 2017). Our findings revealed that the frequency of the derivative allele was relatively close (0.434) to that reported in a prior study (0.495) within the Iranian population (Rokni et al., 2022).

Increased endocytosis, or changes in gene expression associated with risk alleles, may explain some of the specific characteristics of haplotype frequencies. In this context, the haplotypes that carried the derivate alleles in rs75603675-A and rs4303795-G were significantly more prevalent in the case group compared to the reference one. As previously mentioned, the spatial modifications provoked by the derivative allele could enhance the endocytosis signal, increasing susceptibility to infection (Calcagnile et al., 2024). Thus, the risk association of these and other polymorphisms in TMPRSS2 is not surprising, given its role in SARS-CoV-2 entry into the host cells and other mechanisms previously mentioned.

The significance of our findings regarding rs4303795 and rs8134378, along with the results from other research groups, was reinforced by the meta-analysis (Tug et al., 2024; Vargas-Alarcon et al., 2020; Rokni et al., 2022). However, some controversies about rs75603675 emerged: our results proposed the derivative allele -A- as the riskier one, while other studies have proposed that the ancestral allele -C- is riskier (Villapalos-García et al., 2022; Rokni et al., 2022). Nevertheless, haplotype analyses indicated that both alleles seem to contribute to infection, highlighting the importance of allele combinations and the remarkable function of *TMPRSS2* in SARS-CoV-2 infection susceptibility (Rokni et al., 2022). The role of allele frequency distribution is a critical point when haplotypes are constructed. Thus, the discrepancies reported among populations warrant further discussion.

### 4.2 Comparison of *TMPRSS2* polymorphisms among populations

The fact that the case group is genetically four times closer to EUR than the reference group (concerning *TMPRSS2* polymorphisms) allows us to speculate that those Mexican Mestizos that have inherited *TMPRSS2* variants from a European ancestor are genetically more susceptible to SARS-Cov-2 infection than those with *TMPRSS2* variants coming from another parental population. This suggestion came about from the MDS plot (Figure 3C), in which EUR and cases were set to the right of the first dimension, while MXL and the reference group are to the left. Nevertheless, the differences in the allele frequencies between Europeans and the studied population could be due to different demographic events that the Mexican Mestizo population experienced in recent history. On one hand, there was at least one population bottleneck during the Spanish conquest (Gomez et al., 2021). On the other, there was a founder effect coming from the southern part of the Iberian Peninsula; a particular diversity melting pot (Alvarez-Topete et al., 2024).

Several studies, including the present one, have demonstrated that these three SNPs (rs75603675, rs4303795, and rs8134378) exhibit the highest frequencies of risk alleles within European populations (Paniri et al., 2021). Therefore, when haplotype frequencies were analysed, SARS-CoV-2 infection risk became more evident, as observed in other studies (Rokni et al., 2022). Disparities in susceptibility and mortality rates have been reported across different ethnicities; Afro-descendants, Native Americans, Asians and Latinos have shown higher susceptibility rates compared to non-Hispanic whites (Sutton et al., 2022). Nonetheless, the European populations were severely affected, even more than Asians, with Italy and Spain ranking as the two worst-hit countries within the European Community (Ceylan, 2020). Although the Mexican Mestizo population is admixed and presents heterogeneous ancestry, high proportions of European lineages have been reported (Alvarez-Topete et al., 2024; Gomez et al., 2021; Santana et al., 2014). In addition, European countries have exhibited the highest frequencies in terms of *TMPRSS2* missense mutations (Zarubin et al., 2020). Such variants were almost five times more frequent among Southern Europeans (where the Mexican ancestral connection is located) than among East Asians, the second Mexican major ancestry due to its relation to Native Americans, where the missense mutations were scant (frequency ≤0.0001) (Zarubin et al., 2020). As mentioned before, certain allelic variations have been associated with elevated transcription (Barash et al., 2020). Likewise, *TMPRSS2* gene variants such as rs2070788, rs464397, rs469390, and rs383510 could affect expression, particularly in lung tissue (Irham et al., 2020). Nonetheless, several SNPs around the TMPRSS2 polymorphisms likely presented LD, suggesting that other SNPs could also modify its expression. Europeans have the highest frequencies of *TMPRSS2*-upregulating variants, whereas Asians have the lowest ones (Irham et al., 2020). Regarding American ancestry, it presented higher frequencies than Asians and lower than Europeans, which could explain our findings. It is important to note that the data used regarding the Americas is represented only by Latino populations (1KGP; https://www.internationalgenome.org/).

When comparing the minor allele frequency (MAF) in rs75603675, the highest frequency has been reported in EUR (0.405) and the lowest was in EAS (0.017). Our reference group showed MAF = 0.200, possibly reflecting the recent admixture 500 years ago (Shelton et al., 2021; Gomez et al., 2021). Queretaro state and other north-central regions of Mexico exhibit higher proportions of European-derived ancestry compared to the southern regions (Gomez et al., 2021; Santana et al., 2014; Sohail et al., 2023). This may explain the high frequencies of the haplotypes AGA, AGG, and AAG found among the case group, which are also prevalent in Europeans. This suggests that such ancestry could confer a certain susceptibility to SARS-CoV-2 infection, as shown during the pandemic (Ceylan, 2020). Indeed, rs75603675 has been identified as a predictor of COVID-19 severity, and Polyphen-2 has conferred a high score (0.815 out of one), suggesting a possibly hazardous role (Villapalos-García et al., 2022; Paniri et al., 2021). Consequently, in populations with low European ancestry (i.e., Iranian and Vietnamese), no contribution from rs75603675 has been reported (Rokni et al., 2022; Nhung et al., 2022). In the Spanish population from Madrid, the rs75603675-C allele has shown an association with COVID-19 severity (Villapalos-García et al., 2022). As mentioned before, the allele frequency distribution of Mexican Mestizos varies in comparison to that of Spanish.

About the other three *TMRSS2* SNPs (rs456298, rs2070788, and rs12329760) for which we did not find any association, the only result published thus far about rs456298 was also conducted in the Mexican Mestizo population (Posadas-Sanchez et al., 2022). In that study, the A-allele appeared as the risk allele with significant association using several models (i.e., additive, co-dominant, and recessive), although we did not find any association. These discrepancies could be associated with the sample size (three times smaller in our study) and also with the reference sample size (almost half the size of the cases group in the previous study) (Posadas-Sanchez et al., 2022). When wanting to detect allele effects, the sizes of case and reference groups are assumed to be similar (Smith and Day, 1984). In the former study, the reference group was obtained from medical staff, and patients were mixed from several hospitals, which could cause a selection bias (Posadas-Sanchez et al., 2022). Because of the recent formation of the Mexican Mestizo population, a homogeneous genetic architecture is not present (Santana et al., 2014; Noris et al., 2012). Thus, the difference found between the two rs456298 studies could be an effect of the population stratification that has been previously reported (Camacho-Mejorado et al., 2015).

Comparable to the results of the present study, no contribution to COVID-19 symptomatology has been found for rs2070788 and rs12329760 in both Mexican Mestizos and Spanish populations (Martínez-Gomez et al., 2024; Fernandez-de-las-Peñas et al., 2022). Similar findings have been reported regarding rs12329760 in Brazilians (Caldeira de Andrade et al., 2022). By contrast, rs2070788 has been linked to an increased risk of death and comorbidities in Brazilians and among symptomatic and intensive care unit patients in Türkiye (Tug et al., 2024; Caldeira de Andrade et al., 2022). While rs12329760 has shown different distributions between cases and the reference group, it has been associated with COVID-19 susceptibility and mortality in Iranian populations (Rokni et al., 2022). Our results should be interpreted cautiously due to the potential of linkage disequilibrium among these three SNPs or differences in their allele frequencies across the studied populations. It is important to note that our study is focused on infection susceptibility, while others have focused on illness severity and progression.

### 4.3 *ACE2*, *ADAM17*, *FURIN*, *IFITM3* and *VDR* did not show contribution to SARS CoV-2 infection susceptibility

The absence of association with SARS-CoV-2 infection susceptibility by polymorphisms studied on *ACE2*, *ADAM17*, *FURIN*, *IFITM3*, and *VDR* remains controversial in light of previous reports. In this setting, various factors could contribute to this discrepancy. First, most of the polymorphisms analysed in this study have been linked more closely to disease severity, progression and complications rather than to infection susceptibility (Coto et al., 2022; Pandey et al., 2024; Schönfelder et al., 2021; Barbosa-Rodrigues et al., 2023). *VDR*, *FURIN*, and *ADAM1*7 polymorphisms have been related to cytokine storm and, in turn, to illness severity and its subsequent outcome (Coto et al., 2022; Balzanelli et al., 2022; Da Silva Correa da Ronda et al., 2024; Pan et al., 2023). Second, these controversies have also been reported across different geographic regions and even within the same country. *IFITM3* variants have not shown any contribution to COVID-19 in European, Middle Eastern, and Asian populations (Ferreira de Araujo et al., 2022; Li et al., 2022; Dobrijevic et al., 2022; Ren et al., 2023). In contrast, this gene has been linked to disease severity in other populations (Jafarpour et al., 2021; Benmansour et al., 2024). These discrepancies suggest that the influence of genetic architecture, particularly population stratification, might be the primary source of bias in genetic association studies (Wacholder et al., 2000).

*ACE2* has been considered a cornerstone in COVID-19 development and other respiratory diseases, although these findings have not been consistent. Studies carried out in German and Spanish populations (rs2285666 and rs2074192) have shown a significant association between COVID-19 and post-COVID symptoms (Fernandez-de-las-Peñas et al., 2022; Wacharapol et al., 2022). In contrast, studies from Spain, Türkiye and those presented in this document did not find any association (Torres-Fuentes et al., 2021; Wacharapol et al., 2022). The sample size and the genetic structure of the population could influence the findings mentioned, among other potential explanations for these discrepancies. Additionally, the present study was focused on the susceptibility to infection with SARS-CoV-2 rather than on the severity of COVID-19 symptoms. Therefore, it is likely that our findings could be biased in this context.

### 4.4 Strengths and limitations

One of the strengths of the present study is our reference population. This population was randomly obtained from samples taken before the SARS-CoV-2 pandemic, accurately reflecting the genetic architecture of the population that comes to the laboratory and minimising certain biases. However, such a population may also represent a weakness, as our cases were not strictly drawn from the same population as this reference. Also, a limitation of our study is that it did not account for comorbidities since BIMODI’s protocols before the pandemic did not include a questionary about comorbidities. Additionally, the absence of data regarding disease severity, symptomatology, and close contact with COVID-19 individuals also represent limitations; therefore, our results should be interpreted in light of these constraints.

## 5 Conclusion

It is likely that in addition to comorbidities and lifestyle, genetic factors impact SARS-CoV-2 infection susceptibility; to study this, the ancestral background has to be considered, particularly in admixed young populations. We found a significant association of three SNPs (separated and in haplotypes) in the *TMPRSS2* gene with SARS-CoV-2 infection susceptibility in Mexican Mestizos and the possible contribution of European ancestry in the heritage of the risk haplotypes. Further studies should be carried out with larger sample sizes and should consider other factors such as comorbidities, individual precautions, and hygiene practices, among others, to support our findings.

## Data Availability

The original contributions presented in the study are included in the article/Supplementary Material, further inquiries can be directed to the corresponding authors.
