# Supplementary material for: Genetic variants in TMPRSS2 influence SARS-CoV-2 infection susceptibility within Mexican Mestizos
Source: Front Genet. 2025 Apr 14;16:1558189. doi: 10.3389/fgene.2025.1558189 (PMC12034715; doi:10.3389/fgene.2025.1558189)
Supplement: Supplementary file 1 [file Table1.docx]

**S1 Table. Genes and their polymorphisms genotyped in case and reference groups.**

| *ACE2* | *TMPRSS2* | *FURIN* | *ADAM17* | *IFITM3* | *VDR* |
| --- | --- | --- | --- | --- | --- |
| rs2074192 g.15564667C>T (C__16163821_10) | rs456298 g.41464824T>A (C___2592026_1_) | rs4932178 g.90868426C>T  C___1244343_10 | rs10495563 g.9522081G>A  C__30463083_20 | rs12252 g.320772A>G  C_175677529_10 | rs1544410 g.47846052C>T  C___8716062_20 |
| rs233575 g.15564843G>A (C___2260459_10) | rs2070788 g.41470061G>A (C___2592038_1_) | rs2071410 g.90877710C>G  C___1244337_1_ | rs12692386 g.9555777A>G  C__31588436_10 | rs34481144 g.320836C>T  C__26288451_10 | rs2228570 g.47879112A>G  C__12060045_20 |
| rs4240157 g.15568841C>T (C__28018196_20) | rs61735794 g.41470664C>T (C__25622367_10) | rs4702 g.90883330G>A  C___8719648_1_ | rs11684747 g.9557042A>G  C___1829895_10 |  |  |
| rs879922 g.15572684C>G (C___8877953_10) | rs12329760 g.41480570C>T (C__25622353_20) |  |  |  |  |
| rs4646156 g.15578920A>T (C___2551619_10) | rs75603675 g.41507982C>A (C_102710002_10) |  |  |  |  |
| rs4646155 g.15579386C>T (C__32336224_10) | rs4303795 g.41508558A>G (C__11382662_10) |  |  |  |  |
| rs2285666 g.15592225C>T (C___2551626_1_) | rs8134378 g.41521831G>A (C___1723094_1_) |  |  |  |  |

*ACE2*, Angiotensin Converting Enzyme 2; *ADAM*, ADAM metallopeptidase domain 17; *FURIN*, Paired Basic Amino Acid Cleaving Enzyme; *IFITM3*, Interferon Induced Transmembrane Protein 3; *VDR*, Vitamin D Receptor; *TMPRSS2* Transmembrane Serine Protease 2.
