# Supplementary figures and images for: Genetic variants in TMPRSS2 influence SARS-CoV-2 infection susceptibility within Mexican Mestizos
Source: Front Genet. 2025 Apr 14;16:1558189. doi: 10.3389/fgene.2025.1558189 (PMC12034715; doi:10.3389/fgene.2025.1558189)

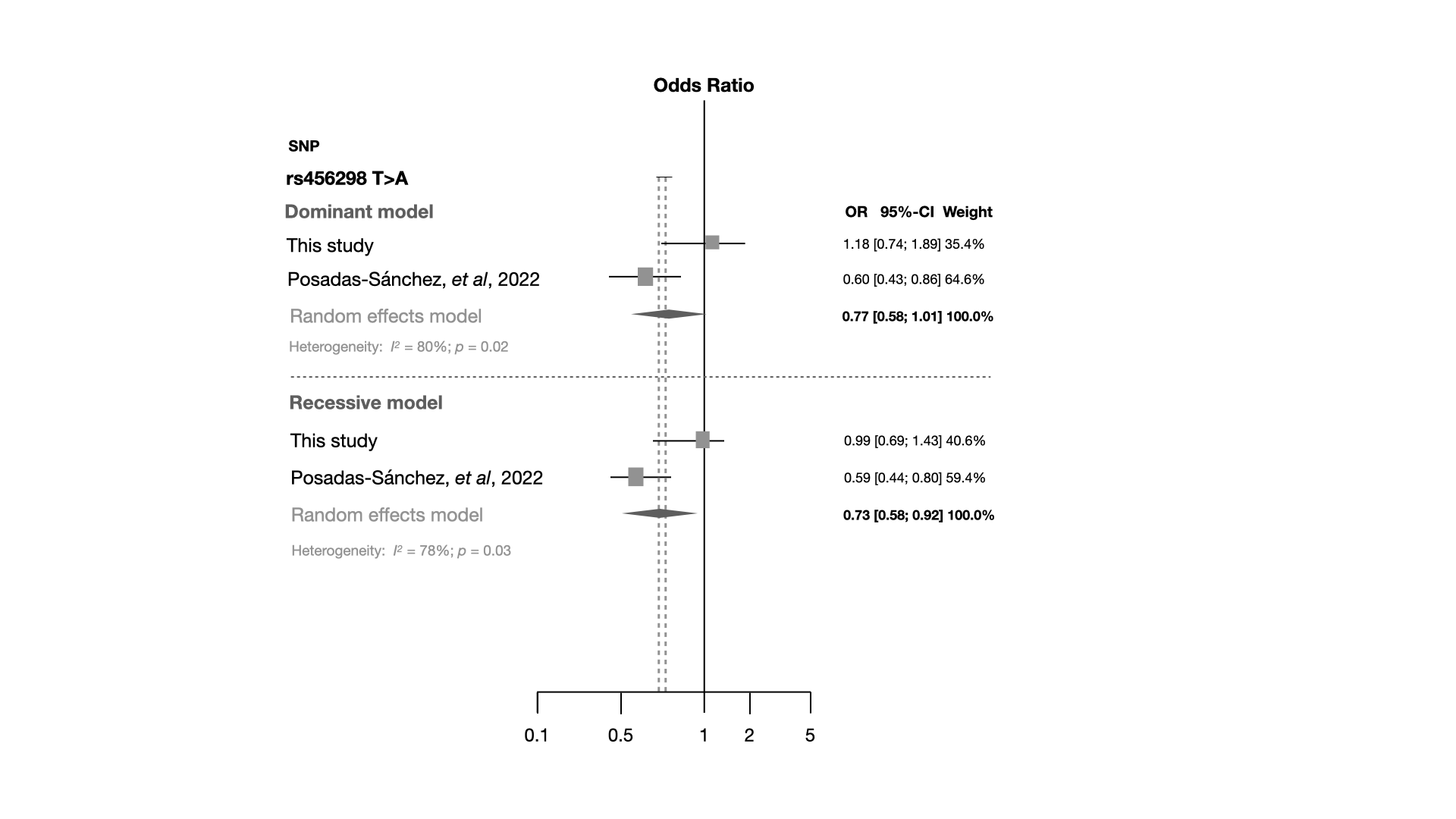

Supplement: Supplementary file 2 [file Image3.tif]

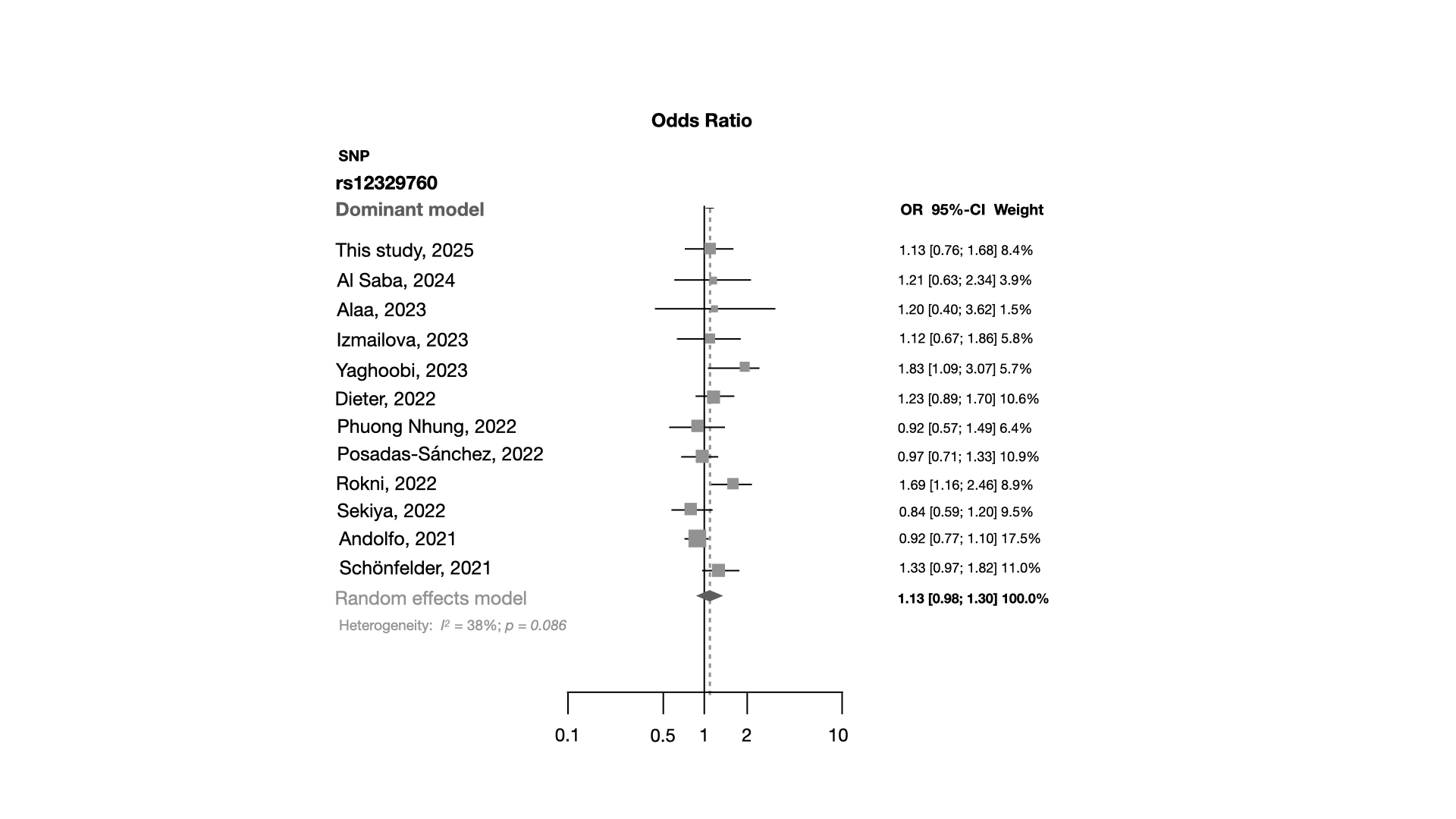

Supplement: Supplementary file 3 [file Image4.tif]

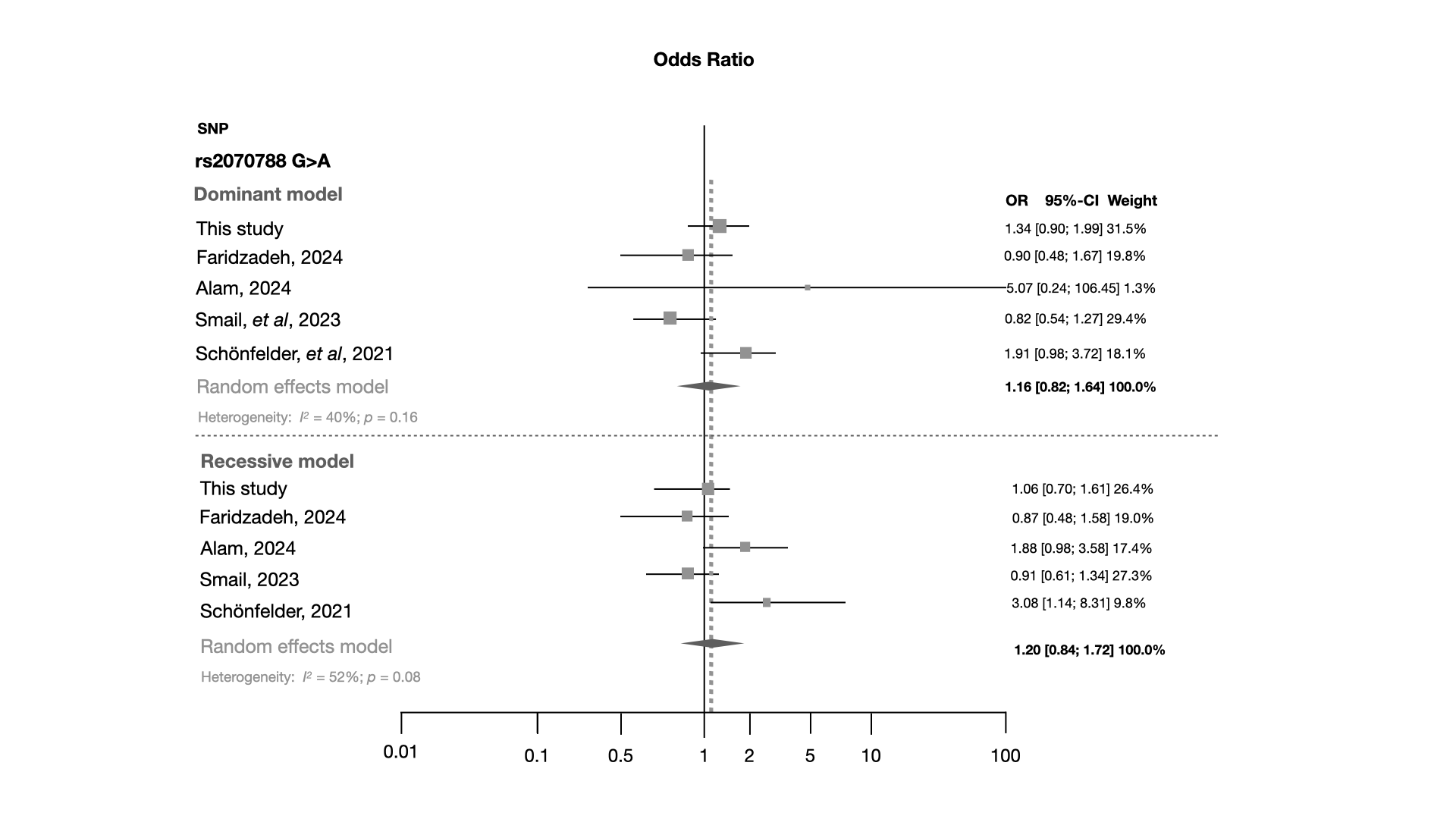

Supplement: Supplementary file 4 [file Image2.tif]

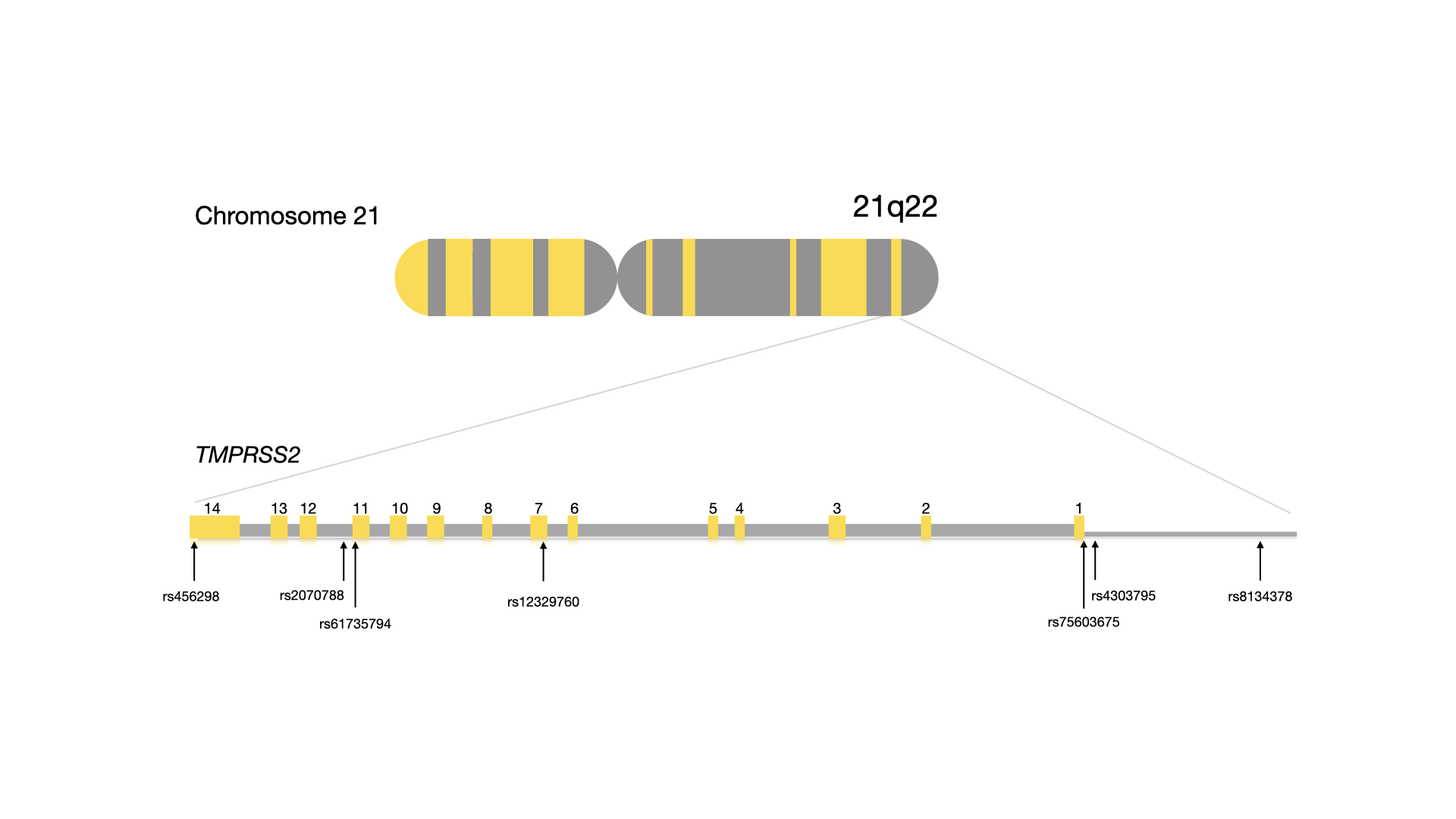

Supplement: Supplementary file 5 [file Image1.tif]

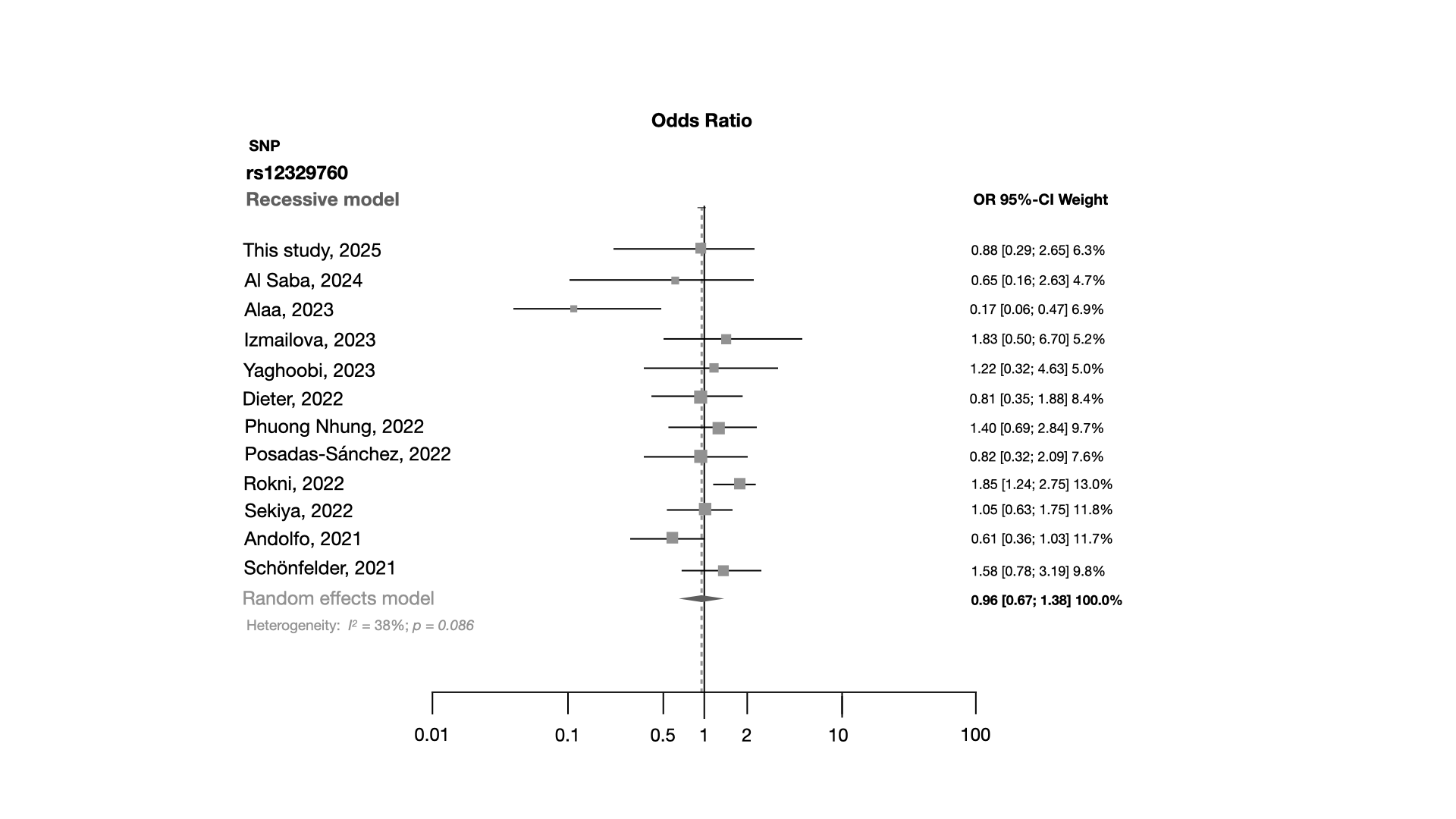

Supplement: Supplementary file 9 [file Image5.tif]
