# Supplementary material for: Genetic variants in TMPRSS2 influence SARS-CoV-2 infection susceptibility within Mexican Mestizos
Source: Front Genet. 2025 Apr 14;16:1558189. doi: 10.3389/fgene.2025.1558189 (PMC12034715; doi:10.3389/fgene.2025.1558189)
Supplement: Supplementary file 6 [file Table2.docx]

**S2 Table. Allele and genotype frequencies, and Hardy-Weinberg equilibrium of the polymorphisms explored in *ADAM17*, *FURIN*, *IFITM3* and *VDR*.**

| Gen | SNP | Case group  (*n*=241) | Reference group  (*n*=248) | MAF | | |
| --- | --- | --- | --- | --- | --- | --- |
|  |  |  |  | **MXL (*n*=64)** | **EUR (*n*=503)** | **EAS (*n*=504)** |
| *ADAM17* | rs10495563 G>A (g.9522081G>A) | | | 0.359 | 0.663 | 0.095 |
|  | G | 332 (0.69) | 345 (0.70) |  |  |  |
|  | A | 150 (0.31) | 151 (0.30) |  |  |  |
|  | GG | 115 (0.48) | 124 (0.50) |  |  |  |
|  | GA | 102 (0.42) | 97 (0.39) |  |  |  |
|  | AA | 24 (0.10) | 27 (0.11) |  |  |  |
|  | HWE *F_IS_* (*p*) | 0.015 (0.48) | 0.078 (0.128) |  |  |  |
|  | rs12692386 A>G (g.9555777A>G) | | | 0.367 | 0.669 | 0.219 |
|  | A | 321 (0.67) | 339 (0.68) |  |  |  |
|  | G | 161 (0.33) | 157 (0.32) |  |  |  |
|  | AA | 108 (0.45) | 120 (0.48) |  |  |  |
|  | AG | 105 (0.44) | 99 (0.40) |  |  |  |
|  | GG | 28 (0.12) | 29 (0.12) |  |  |  |
|  | HWE *F_IS_* (*p*) | 0.023 (0.399) | 0.079 (0.122) |  |  |  |
|  | rs11684747 A>G (g.9557042A>G) | | | 0.117 | 0.188 | 0.020 |
|  | A | 430 (0.89) | 437 (0.88) |  |  |  |
|  | G | 52 (0.11) | 59 (0.12) |  |  |  |
|  | AA | 190 (0.79) | 192 (0.77) |  |  |  |
|  | AG | 50 (0.21) | 53 (0.21) |  |  |  |
|  | GG | 1 (0.00) | 3 (0.01) |  |  |  |
|  | HWE *F_IS_* (*p*) | -0.076 (0.967) | -0.018 (0.713) |  |  |  |
| *FURIN* | rs4932178 C>T (g.90868426C>T) | | | 0.164 | 0.356 | 0.160 |
|  | C | 400 (0.83) | 423 (0.85) |  |  |  |
|  | T | 82 (0.17) | 73 (0.15) |  |  |  |
|  | CC | 167 (0.69) | 183 (0.74) |  |  |  |
|  | CT | 66 (0.27) | 57 (0.23) |  |  |  |
|  | TT | 8 (0.03) | 8 (0.03) |  |  |  |
|  | HWE *F_IS_* (*p*) | 0.032 (0.374) | 0.086 (0.141) |  |  |  |
|  | rs2071410 C>G (g.90877710C>G) | | | 0.148 | 0.323 | 0.059 |
|  | C | 417 (0.87) | 425 (0.86) |  |  |  |
|  | G | 65 (0.13) | 71 (0.14) |  |  |  |
|  | CC | 180 (0.75) | 185 (0.75) |  |  |  |
|  | CG | 57 (0.24) | 55 (0.22) |  |  |  |
|  | GG | 4 (0.02) | 8 (0.03) |  |  |  |
|  | HWE *F_IS_* (*p*) | -0.012 (0.655) | 0.098 (0.113) |  |  |  |
|  | rs4702 G>A (g.90883330G>A) | | | 0.508 | 0.563 | 0.525 |
|  | G | 207 (0.43) | 187 (0.38) |  |  |  |
|  | A | 275 (0.57) | 309 (0.62) |  |  |  |
|  | GG | 40 (0.17) | 40 (0.16) |  |  |  |
|  | GA | 127 (0.53) | 107 (0.43) |  |  |  |
|  | AA | 74 (0.31) | 101 (0.41) |  |  |  |
|  | HWE *F_IS_* (*p*) | -0.073 (0.887) | 0.084 (0.113) |  |  |  |
| *IFITM3* | rs12252 A>G (g.320772A>G) | | | 0.219 | 0.041 | 0.528 |
|  | A | 395 (0.82) | 386 (0.78) |  |  |  |
|  | G | 87 (0.18) | 110 (0.22) |  |  |  |
|  | AA | 160 (0.66) | 156 (0.63) |  |  |  |
|  | AG | 75 (0.31) | 74 (0.30) |  |  |  |
|  | GG | 6 (0.02) | 18 (0.07) |  |  |  |
|  | HWE *F_IS_* (*p*) | -0.050 (0.835) | 0.138 (0.023) |  |  |  |
|  | rs34481144 C>T (g.320836C>T) | | | 0.180 | 0.462 | 0.006 |
|  | C | 382 (0.79) | 385 (0.78) |  |  |  |
|  | T | 100 (0.21) | 111 (0.22) |  |  |  |
|  | CC | 152 (0.63) | 152 (0.61) |  |  |  |
|  | CT | 78 (0.32) | 81 (0.33) |  |  |  |
|  | TT | 11 (0.05) | 15 (0.06) |  |  |  |
|  | HWE *F_IS_* (*p*) | 0.018 (0.478) | 0.062 (0.223) |  |  |  |
| *VDR* | rs1544410 C>T (g.47846052C>T) | | | 0.195 | 0.404 | 0.064 |
|  | C | 375 (0.78) | 399 (0.80) |  |  |  |
|  | T | 107 (0.22) | 97 (0.20) |  |  |  |
|  | CC | 151 (0.63) | 164 (0.66) |  |  |  |
|  | CT | 76 (0.30) | 71 (0.29) |  |  |  |
|  | TT | 17 (0.07) | 13 (0.05) |  |  |  |
|  | HWE *F_IS_* (*p*) | 0.125 (0.035) | 0.092 (0.125) |  |  |  |
|  | rs2228570 A>G (g.47879112A>G) | | | 0.516 | 0.622 | 0.582 |
|  | A | 211 (0.44) | 230 (0.46) |  |  |  |
|  | G | 271 (0.56) | 266 (0.54) |  |  |  |
|  | AA | 46 (0.19) | 53 (0.21) |  |  |  |
|  | AG | 119 (0.49) | 124 (0.50) |  |  |  |
|  | GG | 76 (0.32) | 71 (0.29) |  |  |  |
|  | HWE *F_IS_* (*p*) | -0.001 (0.541) | -0.003 (0.606) |  |  |  |

All SNPs exhibit the reference SNP cluster identification (rs), followed by the standard nomenclature of the Human Genome Variation Society (HGVS) in between brackets *ADAM*, ADAM metallopeptidase domain 17; EAS, East Asian populations; EUR European populations; *F_IS_*, Fixation index; *FURIN*, Paired Basic Amino Acid Cleaving Enzyme; HWE, Hardy-Weinberg Equilibrium, *IFITM3*, Interferon Induced Transmembrane Protein 3; MAF, Minimum Allele Frequency; MXL, Mexican ancestry in Los Angeles California; *n,* number of samples; *p*, p-value; *VDR*; Vitamin D Receptor.
