# Supplementary material for: Genetic variants in TMPRSS2 influence SARS-CoV-2 infection susceptibility within Mexican Mestizos
Source: Front Genet. 2025 Apr 14;16:1558189. doi: 10.3389/fgene.2025.1558189 (PMC12034715; doi:10.3389/fgene.2025.1558189)
Supplement: Supplementary file 7 [file Table3.docx]

**S3 Table. Allele and genotype frequencies of the polymorphisms explored in *ACE2* gene.**

| SNP | Case group | | Reference group | | MAF | | | | | | | | |
| --- | --- | --- | --- | --- | --- | --- | --- | --- | --- | --- | --- | --- | --- |
|  |  |  |  |  | **MXL** | | | **EUR** | | | **EAS** | | |
|  | Female (*n*=134) | Male  (*n*=107) | Female (*n=*124) | Male (*n=*124) | Female  (*n=*32) | Male  (*n=*32) | Female (*n=*263) | | Male (*n=*240) | Female (*n=*260) | | Male (*n=*244) |  |
| rs2074192 C>T (g.15564667C>T) | | | | | 0.36 | 0.47 | 0.41 | | 0.45 | 0.43 | | 0.43 |  |
| C | 151 (0.56) | 63 (0.59) | 131 (0.53) | 73 (0.59) |  |  |  |  |  |  |  |  |  |
| T | 117 (0.44) | 44 (0.41) | 117 (0.47) | 51 (0.41) |  |  |  |  |  |  |  |  |  |
| CC | 50 (0.37) |  | 35 (0.28) |  |  |  |  |  |  |  |  |  |  |
| CT | 51 (0.38) |  | 61 (0.49) |  |  |  |  |  |  |  |  |  |  |
| TT | 33 (0.25) |  | 28 (0.23) |  |  |  |  |  |  |  |  |  |  |
| rs233575 G>A (g.15564843G>C) | | | | | 0.20 | 0.13 | 0.36 | | 0.28 | 0.01 | | 0 |  |
| G | 58 (0.22) | 20 (0.19) | 39 (0.16) | 23 (0.19) |  |  |  |  |  |  |  |  |  |
| A | 210 (0.78) | 87(0.81) | 209 (0.84) | 101 (0.81) |  |  |  |  |  |  |  |  |  |
| GG | 86 (0.64) |  | 4 (0.03) |  |  |  |  |  |  |  |  |  |  |
| GA | 38 (0.28) |  | 31 (0.25) |  |  |  |  |  |  |  |  |  |  |
| AA | 10 (0.07) |  | 89 (0.72) |  |  |  |  |  |  |  |  |  |  |
| rs4240157 C>T (g.15568841C>T) | | | | | 0.23 | 0.16 | 0.39 | | 0.29 | 0.04 | | 0.03 |  |
| C | 61 (0.23) | 24 (0.22) | 45 (0.18) | 29 (0.23) |  |  |  |  |  |  |  |  |  |
| T | 207 (0.77) | 83 (0.78) | 203 (0.82) | 95 (0.77) |  |  |  |  |  |  |  |  |  |
| CC | 9 (0.07) |  | 5 (0.04) |  |  |  |  |  |  |  |  |  |  |
| CT | 43 (0.32) |  | 35 (0.28) |  |  |  |  |  |  |  |  |  |  |
| TT | 82 (0.61) |  | 84(0.68) |  |  |  |  |  |  |  |  |  |  |
| rs879922 C>G (g.15572684C>G) | | | | | 0.23 | 0.16 | 0.38 | | 0.30 | 0.04 | | 0.03 |  |
| C | 60 (0.22) | 24 (0.22) | 35 (0.14) | 30 (0.24) |  |  |  |  |  |  |  |  |  |
| G | 208 (0.78) | 83 (0.78) | 213 (0.86) | 94 (0.76) |  |  |  |  |  |  |  |  |  |
| CC | 10 (0.07) |  | 5 (0.04) |  |  |  |  |  |  |  |  |  |  |
| CG | 40 (0.30) |  | 25 (0.20) |  |  |  |  |  |  |  |  |  |  |
| GG | 84 (0.63) |  | 94 (0.76) |  |  |  |  |  |  |  |  |  |  |
| rs4646156 A>T (g.15578920A>T) | | | | | 0.19 | 0.13 | 0.38 | | 0.28 | 0.01 | | 0 |  |
| A | 51 (0.19) | 18 (0.17) | 36 (0.15) | 28 (0.23) |  |  |  |  |  |  |  |  |  |
| T | 217 (0.81) | 89 (0.83) | 212 (0.85) | 96 (0.77) |  |  |  |  |  |  |  |  |  |
| AA | 4 (0.03) |  | 5 (0.04) |  |  |  |  |  |  |  |  |  |  |
| AT | 43 (0.32) |  | 26 (0.21) |  |  |  |  |  |  |  |  |  |  |
| TT | 87 (0.65) |  | 93 (0.75) |  |  |  |  |  |  |  |  |  |  |
| rs4646155 C>T (g.15579386C>T) | | | | | 0.02 | 0 | 0 | | 0 | 0.03 | | 0.03 |  |
| C | 266 (0.99) | 106 (0.99) | 248 (1) | 123 (0.99) |  |  |  |  |  |  |  |  |  |
| T | 2 (0.01) | 1 (0.01) | 0 (0) | 1 (0.01) |  |  |  |  |  |  |  |  |  |
| CC | 132 (0.99) |  | 124 (1) |  |  |  |  |  |  |  |  |  |  |
| CT | 2 (0.01) |  | 0 (0) |  |  |  |  |  |  |  |  |  |  |
| TT | 0 (0) |  | 0 (0) |  |  |  |  |  |  |  |  |  |  |
| rs2285666 C>T (g.15592225C>T) | | | | | 0.39 | 0.31 | 0.23 | | 0.25 | 0.54 | | 0.53 |  |
| C | 169 (0.63) | 63 (0.59) | 135 (0.62) | 80 (0.65) |  |  |  |  |  |  |  |  |  |
| T | 99 (0.37) | 44 (0.41) | 95 (0.38) | 44 (0.35) |  |  |  |  |  |  |  |  |  |
| CC | 55 (0.41) |  | 46 (0.37) |  |  |  |  |  |  |  |  |  |  |
| CT | 59 (0.44) |  | 61 (0.49) |  |  |  |  |  |  |  |  |  |  |
| TT | 20 (0.15) |  | 17 (0.14) |  |  |  |  |  |  |  |  |  |  |

All SNPs exhibit the reference SNP cluster identification (rs), followed by the standard nomenclature of the Human Genome Variation Society (HGVS) in between brackets. *ACE2*, Angiotensin Converting Enzyme 2; EAS, East Asian populations; EUR European populations; MAF, Minimum Allele Frequency; MXL, Mexican ancestry in Los Angeles California; *n,* number of samples.
