# Supplementary material for: Genetic variants in TMPRSS2 influence SARS-CoV-2 infection susceptibility within Mexican Mestizos
Source: Front Genet. 2025 Apr 14;16:1558189. doi: 10.3389/fgene.2025.1558189 (PMC12034715; doi:10.3389/fgene.2025.1558189)
Supplement: Supplementary file 8 [file Table4.docx]

**S4 Table. Matrix of genetic distance values (*F_ST_*, below the diagonal) regarding the SNPs rs75603675, rs4303795, and rs8134378 located on the *TMPRSS2* gene and the *p*-values (above the diagonal) among the case group, the reference group, MXL and EUR data from the 1000 genome project.**

| *F_ST_ \ p* | Case group | Reference group | MXL | EUR |
| --- | --- | --- | --- | --- |
| Case group | * | **≤0.0001** | 0.42342 | **≤0.0001** |
| Control group | 0.2684 | * | 0.09730 | **≤0.0001** |
| MXL | -0.00147 | 0.01162 | * | **≤0.0001** |
| EUR | 0.01776 | 0.08148 | 0.03018 | * |

The *p*-value was corrected by the false discovery rate (FDR). EUR, European populations; *F_ST_*, Genetic distance; MXL, Mexican Ancestry in Los Angeles California; *p*, p-value.
